# Association of Sex or Race With the Effect of Weight Loss on Physical Function: A Secondary Analysis of 8 Randomized Clinical Trials

**DOI:** 10.1001/jamanetworkopen.2020.14631

**Published:** 2020-08-21

**Authors:** Kristen M. Beavers, Rebecca H. Neiberg, Stephen B. Kritchevsky, Barbara J. Nicklas, Dalane W. Kitzman, Stephen P. Messier, W. Jack Rejeski, Jamy D. Ard, Daniel P. Beavers

**Affiliations:** 1 Department of Health and Exercise Science, Wake Forest University, Winston-Salem, North Carolina; 2 Department of Department of Biostatistics and Data Science, Wake Forest School of Medicine, Winston-Salem, North Carolina; 3 Sections of Gerontology and Cardiovascular Medicine, Department of Internal Medicine, Wake Forest School of Medicine, Winston-Salem, North Carolina; 4 Department of Epidemiology and Prevention, Wake Forest School of Medicine, Winston-Salem, North Carolina

## Abstract

**Importance:**

Consideration of differential treatment effects among subgroups in clinical trial research is a topic of increasing interest. This is an especially salient issue for weight loss trials.

**Objective:**

To determine whether stratification by sex and race is associated with meaningful differences in physical function response to weight loss among older adults.

**Design, Setting, and Participants:**

This pooled analysis used individual-level data from 8 completed randomized clinical trials of weight loss conducted at Wake Forest University or Wake Forest School of Medicine, Winston-Salem, North Carolina. Data were housed within the Wake Forest Older Americans Independence Center data repository and provided complete exposure, outcome, and covariate information. Data were collected from November 1996 to March 30, 2017, and analyzed from August 15, 2019, to June 10, 2020.

**Exposures:**

Treatment arms within each study were collapsed into caloric restriction (CR [n = 734]) and non-CR (n = 583) categories based on whether caloric restriction was specified in the original study protocol.

**Main Outcomes and Measures:**

Objectively measured 6-month change in weight, fast-paced gait speed (meters per second), and Short Physical Performance Battery (SPPB) score.

**Results:**

A total of 1317 adults (mean [SD] age, 67.7 [5.4] years; 920 [69.9%] female; 275 [20.9%] Black) with overweight or obesity (mean [SD] body mass index [calculated as weight in kilograms divided by height in meters squared], 33.9 [4.4]) were included at baseline. Six-month weight change achieved among those randomized to CR was −7.7% (95% CI, −8.3% to −7.2%), with no difference noted by sex; however, White individuals lost more weight than Black individuals assigned to CR (−9.0% [95% CI, −9.6% to −8.4%] vs −6.0% [95% CI, −6.9% to 5.2%]; *P* < .001), and all CR groups lost a significantly greater amount from baseline compared with non-CR groups (Black participants in CR vs non-CR groups, −5.3% [95% CI, −6.4% to −4.1%; *P* < .001]; White participants in CR vs non-CR groups, −7.2% [95% CI, −7.8% to −6.6%; *P* < .001]). Women experienced greater weight loss–associated improvement in SPPB score (CR group, 0.35 [95% CI, 0.18-0.52]; non-CR group, 0.08 [95% CI, −0.11 to 0.27]) compared with men (CR group, 0.23 [95% CI, 0.00-0.46]; non-CR group, 0.34 [95% CI, 0.09-0.58]; *P* = .03). Black participants experienced greater weight loss–associated improvement in gait speed (CR group, 0.08 [95% CI, 0.05-0.10] m/s; non-CR group, 0.02 [95% CI, −0.01 to 0.05] m/s) compared with White participants (CR group, 0.07 [95% CI, 0.06-0.09] m/s; non-CR group, 0.06 [95% CI, 0.04-0.08] m/s; *P* = .02).

**Conclusions and Relevance:**

The association of weight loss on physical function in older adults appears to differ by sex and race. These findings affirm the need to consider biological variables in clinical trial design.

## Key Points

**Question:**

Is sex or race associated with the physical function response to a weight loss intervention among older adults?

**Findings:**

In this pooled secondary analysis of 1317 individuals participating in 8 randomized clinical trials of weight loss, including 397 (30.1%) men and 275 (20.9%) Black participants, greater weight loss–associated improvement in short physical performance battery score was observed in women vs men, and greater gait speed improvement in Black vs White participants.

**Meaning:**

These findings suggest that the benefits of weight loss on physical function in older adults differ by sex and race, underscoring the need to consider relevant biological variables in clinical research design.

## Teaser

This secondary analysis of 8 randomized clinical trials assesses whether stratification by sex and race is associated with meaningful differences in physical function response to weight loss among older adults.

## Introduction

Consideration of relevant biological variables, such as sex and race, in clinical research is a topic of increasing interest.^1^ Research funded by—and intended to benefit—the public should be all inclusive, and implementation of effective evidence-based medicine depends on appropriate representation of diverse groups in research studies.^2^ Disparities in health and longevity are well documented,^3,4^ and intervention efficacy can vary significantly by subgroup. For instance, results associated with many of the compounds tested through the Interventions Testing Program (a National Institute on Aging–sponsored study investigating treatments to extend lifespan in mice) demonstrate major discrepancies by sex.^5^ Analyses from the cardiology literature also show discordant pharmacological treatment effects in men vs women^6^ and Black vs White individuals.^7^ Although acceptance of federal research policies designed to enhance diversity among clinical trial participants is increasing, negative attitudes regarding practical implementation persist,^8^ with few randomized clinical trials sufficiently designed to delineate treatment effects by sex and race subgroups, despite a growing call to action.^9^

Appropriate representation of diverse populations in clinical trials is an especially salient issue for weight loss interventions. Although data show that White men and women are equally affected by obesity,^10^ men are historically underrepresented in weight loss trials.^11^ Similarly, non-Hispanic Black individuals—for whom obesity is more prevalent compared with their White counterparts^10^—typically constitute less than 20% of all study participants.^12^ Furthermore, responsivity to weight loss interventions is variable, and data indicate that men and minorities respond differently than non-Hispanic White women (who represent most weight loss study participants). For example, men tend to lose more weight than women when given the same weight loss intervention.^11^ Conversely, non-Hispanic Black participants typically lose 2 to 3 kg less than non-Hispanic White participants in the same 6- to 12-month behavioral weight loss intervention; yet interestingly, cardiometabolic risk factor modification appears similar across groups.^13^ This observation implies that the magnitude of weight loss may interact with race/ethnicity to influence health outcomes and raises the question whether weight loss recommendations should be subgroup specific.

During the past decade, a number of weight loss trials were devoted to testing the effects of diet and exercise on physical function among older adults with obesity.^14^ More than one-third of US residents 65 years and older report some degree of physical disability,^15^ which can result in dependency, institutionalization, and high rates of use of health care services.^16^ Although obesity has a strong association with disability onset,^17^ Black individuals are more likely to have^18^ or develop^19^ physical impairment than White individuals for the same body mass index (BMI). Women also display a greater susceptibility to obesity-associated disability than men^20^; however, older women preserve physical capacity better than men over time,^21^ which may translate into differential patterns of treatment response. As with the larger weight loss literature, underrepresentation of men and minorities in aging research makes it challenging to establish whether physical function responses to intentional weight loss are generalizable across all groups.

Data collected through the Wake Forest Older Americans Independence Center, Winston-Salem, North Carolina, provide a unique opportunity to address this important question. Individual participant level data from 1317 middle-aged and older adults (397 male [30.1%] and 275 Black [20.9%]) enrolled in 8 weight loss interventions who completed a standardized physical function assessment at baseline and 6-month follow-up provide an infrastructure to examine whether sex and race are associated with the effect of weight loss on physical function. We hypothesize that stratification by sex and race will reveal meaningful differences in aggregate treatment responses.

## Methods

### Study and Participants Descriptions

Individual participant data from 8 randomized clinical trials of weight loss conducted at Wake Forest University or Wake Forest School of Medicine and housed within the Wake Forest Older Americans Independence Center data repository were eligible for inclusion in the pooled analysis. All studies assessed common measures of physical function before and 6 months after assignment to a caloric restriction (CR) intervention or to a non-CR control condition, with or without exercise. The Wake Forest Health Sciences institutional review board approved secondary analyses pertaining to the pooled project. All participants provided written informed consent to participate in the 8 trials. We followed the Preferred Reporting Items for Systematic Reviews and Meta-analyses (PRISMA) reporting guideline.

Data were acquired from November 1996 to March 30, 2017. Primary outcome studies from included trials, including study design details, have been published.^22,23,24,25,26,27,28,29^ Table 1 provides a brief description of each study (ordered by study acronym), including sample size, distribution by sex and race, age, health status, intervention strategy (and associated sample size), and available physical function data. Of the 1590 baseline visits across all studies, 1382 had a 6-month follow-up visit, and 1359 had 6-month weight change data. Of these participants, 42 were excluded from the primary analysis owing to missing at least 1 covariate (race [n = 17], educational level [n = 8], diabetes status [n = 14], hypertension status [n = 15], and cardiovascular disease [n = 6]; some individuals were missing >1 covariate), yielding the final sample of 1317 participants.

**Table 1.** Descriptive Summary of Weight Loss RCTs Included in the Pooled Analysis

Source (trial name)	No. of participants	Male, No. (%)	Black, No. (%)	Mean age, y	Health status	Intervention (No. of participants)	No. with complete gait speed	No. with complete SPPB
Messier et al,^22^ 2004 (ADAPT)	228	66 (28)	53 (23)	69	Overweight/obese; OA	CR (n = 62); AE (n = 57); CR plus AE (n = 53); control (n = 56)	200	0
Normandin et al, 2018^28^ (APPLE) (PI, Nicklas)	33	8 (24)	6 (18)	70	Obese; OA	CR (n = 15) CR plus vest (n = 18)^a^	33	33
Rejeski et al,^23^ 2011 (CLIP)	262	88 (34)	44 (17)	67	Overweight/obese; CVD/METS	CR plus AE (n = 95); AE (n = 83); control (n = 84)	257	256
Nicklas et al, 2015^25^ (I’M FIT)	110	50 (45)	13 (12)	70	Overweight/obese; at-risk for disability	CR plus RE (n = 55); RE (n = 55)	109	110
Messier et al, 2013^24^ (IDEA)	356	106 (30)	62 (17)	66	Overweight/obese; OA	CR (n = 107); AE (n = 118); CR plus AE (n = 131)	338	131
Nicklas et al,^26^ 2019 (INFINITE)	154	39 (25)	36 (23)	69	Obese	Low CR plus AE (n = 58); high CR plus AE (n = 52) AE (n = 44)	140	153
Beavers et al,^29^ 2019 (Medifast)^b^	81	22 (27)	20 (25)	70	Obese/at-risk for disability	CR (n = 42); control (n = 39)	79	81
Kitzman et al,^27^ 2016 (SECRET)	93	18 (19)	41 (44)	67	Overweight/obese; HFPEF	CR (n = 24); AE (n = 24); CR plus AE (n = 22); control (n = 23)	89	89

Abbreviations: ADAPT, Arthritis, Diet, and Activity Promotion Trial; AE, aerobic exercise; APPLE, Arthritis Pilot for Preserving Muscle While Losing Weight; CLIP, Cooperative Lifestyle Intervention Program; CR, caloric restriction; CVD/METS, cardiovascular disease or metabolic syndrome; HFPEF, heart failure with preserved ejection fraction; I’M FIT, Improving Muscle for Functional Independence Trial; IDEA, Intensive Diet and Exercise for Improving Knee Osteoarthritis in Obese and Overweight Older Adults; INFINITE, Investigating Fitness Interventions in the Elderly; OA, osteoarthritis; PI, principal investigator; RCT, randomized clinical trial; RE, resistance exercise; SECRET, Study of the Effect of Caloric Restriction and Exercise Training in Patients With Heart Failure and a Normal Ejection Fraction; SPPB, Short Physical Performance Battery.

^a^ Intervention included weighted vest use during activities of daily living.

^b^ Effect of High Protein Weight Loss for Seniors study using the Medifast plan.

### Exposure Measures: CR and Weight Change Categories

For the primary analysis, arms within each study were collapsed into CR (n = 734) and non-CR (n = 583) categories based on whether weight loss via CR was specified in the original study protocol. Among 13 study-specific arms collapsed into the CR arm, 5 included participants randomized to CR only (n = 250), and 8 included participants randomized to CR combined with exercise (n = 484). Among 10 study-specific arms collapsed into the non-CR arm, 4 included participants randomized to attention control (n = 202), and 6 included participants randomized to exercise only (n = 381). Categorical amount of weight change from baseline to 6 months (weight gain/stability, <3% loss [n = 596]; moderate weight loss, 3%-7% [n = 262]; and high weight loss, ≥7% [n = 459]) was used as a secondary exposure variable among all participants.

### Outcome Measures: Objectively Measured Physical Function

All physical function measures were assessed by trained and blinded assessors, using standardized protocols at baseline and 6 months. All studies collected fast-paced gait speed, and 7 of the 8 studies (excluding the Arthritis, Diet, and Activity Promotion Trial^22^) included the Short Physical Performance Battery (SPPB). Time recorded from the 6-minute walk (685 [52.0%] of the study sample) or fast-paced 400-m walk (632 [48.0%]) was used to derive fast-paced gait speed. During the 6-minute walk test,^30^ participants were asked to walk as far as they could around a circular track in 6 minutes. During the 400-m walk test,^31^ participants were asked to walk 10 laps of a 40-m course and were given a maximum of 15 minutes to complete the test. The SPPB is a standardized measure of physical performance that assesses standing balance, usual gait velocity for a 4-m course, and time to sit down and rise from a chair 5 times as quickly as possible.^32^ Each task is scored on a scale of 0 to 4, with 0 indicating the inability to complete the task and 1 to 4 indicating the level of performance. The total SPPB score ranges from 0 (lowest function) to 12 (highest function).

### Covariate and Exploratory Measures

All studies captured self-reported demographic characteristics (age, sex, race, and educational level) and presence of select comorbidities (diabetes, hypertension, or cardiovascular disease) via questionnaire at baseline. Specifically for sex and race, participants selected options defined by the investigator. For the present pooled analysis, sex was categorized as male or female, and the National Institutes of Health race format was used to categorize individuals as Black or African American (henceforth referred to as Black) or White. Standing height was measured using a clinical stadiometer, and BMI was measured with a standard scale (with shoes and outer garments removed). Body mass index was calculated as weight in kilograms divided by height in meters squared. Baseline and follow-up whole-body fat and lean mass were also measured in 4 studies using dual-energy x-ray absorptiometry (DXA) on the same machine (Hologic Discovery) and following a standardized protocol.^25,26,27,28^

### Statistical Analysis

Data were analyzed from August 15, 2019, to June 10, 2020. Baseline data were analyzed using descriptive statistics, with means and SDs computed for continuous variables and counts and proportions for discrete variables. The primary analytic model sample sizes differed by outcome measure (percentage weight change [n = 1317], gait speed [n = 1245], and SPPB [n = 853]). Six-month pooled treatment effects on weight, gait speed, and SPPB score by group were estimated using general linear models adjusted for age, sex, race, study, educational level, BMI (gait speed and SPPB models only), comorbid status, and baseline value of the outcome. Tests of heterogeneity of change were first examined among CR or weight loss category, sex, and race as a 3-way interaction term. Subsequently, heterogeneity between CR or weight loss category and sex or race were then investigated through 2-way interaction terms. Nonsignificant interactions were dropped from the models to generate final estimates. Sensitivity analyses examining the potential influence of exercise were performed by: (1) testing 3-way interactions among CR or weight loss category, sex or race, and a binary indicator for exercise assignment (if structured exercise was included in the original study protocol); and (2) including the binary indicator for exercise assignment as a covariate in significant 2-way interaction term models. Last, exploratory analyses using change in total body fat and lean mass as outcome measures were conducted on the subset of participants with DXA data collected at baseline and 6-month visits (n = 360). All analyses were generated using SAS software, version 9.4 (SAS Institute, Inc), using 2-sided hypothesis tests and assuming a type I error rate of 0.05 for all comparisons. *P* < .05 indicated significance.

## Results

### Participant Characteristics

Table 2 presents baseline characteristics for the participants included in the pooled study sample, by CR and weight change category. Overall, participants had a mean age of 67.7 (5.4) years and had class I obesity (mean BMI, 33.9 [4.4]). A total of 920 participants (69.9%) were women and 397 (30.1%) were men; 275 were Black (20.9%). No differences in any baseline characteristic were noted by CR category, except SPPB score, which was higher in the CR group (10.5 [1.4] vs 10.3 [1.6]; *P* = .01). Similar uniformity was noted when stratifying by weight change category, with only 2 significant differences noted: those in the high weight loss category were more likely to be White (394 of 459 [85.8%] vs 65 of 459 [14.2%]; *P* < .001) and presented with a faster mean baseline gait speed (1.3 [0.2] vs 1.2 [0.2] m/s; *P* = .02). Participant characteristics, stratified by sex and race, are also presented in eTables 1 and 2 in the Supplement. Baseline characteristics of the subset of participants with DXA (n = 360) showed that compared with the sample without DXA, they were more likely to be Black (88 of 360 [24.4%] vs 187 of 957 [19.5%]) and have diabetes (70 of 360 [19.4%] vs 118 of 957 [12.3%]) or hypertension (231 of 360 [64.2%] vs 537 of 957 [56.1%]).

**Table 2.** Demographic Characteristics by Caloric Restriction and Weight Change Category

Variable	Treatment group^a^		Weight change category^a^^,^^b^		
	Non-CR (n = 583)	CR (n = 734)	Gain/stability (n = 596)	Moderate loss (n = 262)	High loss (n = 459)
Age, mean (SD), y	67.6 (5.3)	67.7 (5.5)	67.6 (5.3)	67.7 (5.7)	67.7 (5.4)
Sex					
Female	399 (68.4)	521 (71.0)	413 (69.3)	188 (71.8)	319 (69.5)
Male	184 (31.6)	213 (29.0)	183 (30.7)	74 (28.2)	140 (30.5)
Race					
White	468 (80.3)	574 (78.2)	450 (75.5)	198 (75.6)	394 (85.8)
Black	115 (19.7)	160 (21.8)	146 (24.5)	64 (24.4)	65 (14.2)
Educational level					
Primary/secondary only	118 (20.2)	137 (18.7)	119 (20.0)	43 (16.4)	93 (20.3)
College graduate	342 (58.7)	438 (59.7)	366 (61.4)	155 (59.2)	259 (56.4)
Postcollege graduate	123 (21.1)	159 (21.7)	111 (18.6)	64 (24.4)	107 (23.3)
BMI, mean (SD)	33.8 (4.7)	34.0 (4.2)	33.8 (4.4)	33.9 (4.5)	34.1 (4.4)
Comorbidities					
Diabetes	89 (15.3)	99 (13.5)	89 (14.9)	41 (15.6)	58 (12.6)
Hypertension	336 (57.6)	432 (58.9)	348 (58.4)	156 (59.5)	264 (57.5)
CVD history	204 (35.0)	244 (33.2)	223 (37.4)	84 (32.1)	141 (30.7)
Physical function assessments					
Fast gait speed, m/s^c^	1.23 (0.23)	1.24 (0.22)	1.22 (0.22)	1.23 (0.22)	1.26 (0.22)
SPPB (0-12 score)^d^	10.27 (1.59)	10.52 (1.39)	10.34 (1.51)	10.30 (1.60)	10.54 (1.40)
DXA body composition measures, mean (SD)^e^					
Total fat mass, kg	37.7 (8.7)	38.2 (8.6)	37.4 (8.4)	38.1 (8.5)	38.6 (9.0)
Total lean mass, kg	53.8 (11.3	52.8 (11.0)	53.7 (11.3)	52.2 (10.4)	53.3 (11.4)
Body fat, %	40.2 (7.2)	41.1 (7.0)	40.2 (7.1)	41.2 (6.7)	41.1 (7.2)

Abbreviations: BMI, body mass index (calculated as weight in kilograms divided by square of height in meters); CVD, cardiovascular disease; DXA, dual energy x-ray absorptiometry; SPPB, short physical performance battery.

^a^ Unless otherwise indicated, data are expressed as number (percentage) of participants.

^b^ Weight gain/stability indicates less than 3% loss; moderate weight loss, 3% to 7%; high weight loss, at least 7%.

^c^ Includes 1245 participants.

^d^ Includes 853 participants. Scores range from 0 to 12, with higher scores indicating highest function.

^e^ Includes 360 participants.

### Overall Treatment Effects on Achieved Weight Loss and Physical Function

In pooled analyses, mean 6-month weight change among CR participants was −7.7% (95% CI, −8.3% to −7.2%), whereas non-CR participants lost a more modest amount of weight (−1.0% [95% CI, −1.6% to −0.3%]; *P* < .001). In general agreement with individual trial findings, pooled physical function treatment effect estimates showed improvements in fast-paced gait speed (0.02 [95% CI, 0.01-0.04] m/s) in the CR vs non-CR groups (*P* = .01). A marginal, albeit nonsignificant, improvement in SPPB score (0.15 [(95% CI, −0.01 to 0.32]; *P* = .06) in the CR vs non-CR groups was also observed.

### Association of Sex or Race With the Effect of Weight Loss on Physical Function

No difference in weight change was noted by sex; however, White individuals lost more weight than Black individuals assigned to CR (−9.0% [95% CI, −9.6% to −8.4%] vs −6.0% [95% CI, −6.9% to −5.2%]; *P* < .001), and all CR groups lost a significantly greater amount from baseline in comparison with the non-CR groups (Black participants in CR vs non-CR groups, −5.3% [95% CI, −6.4% to −4.1%; *P* < .001]; White participants in CR vs non-CR groups, −7.2% [95% CI, −7.8% to −6.6%; *P* < .001]). No significant 3-way interactions were observed among CR or weight change category, sex, and race; thus, 2-way interaction term models were pursued. Three significant (*P* < .05) 2-way interaction terms were observed between sex or race and CR or weight change category, with stratified results presented in the Figure.

**Figure.**
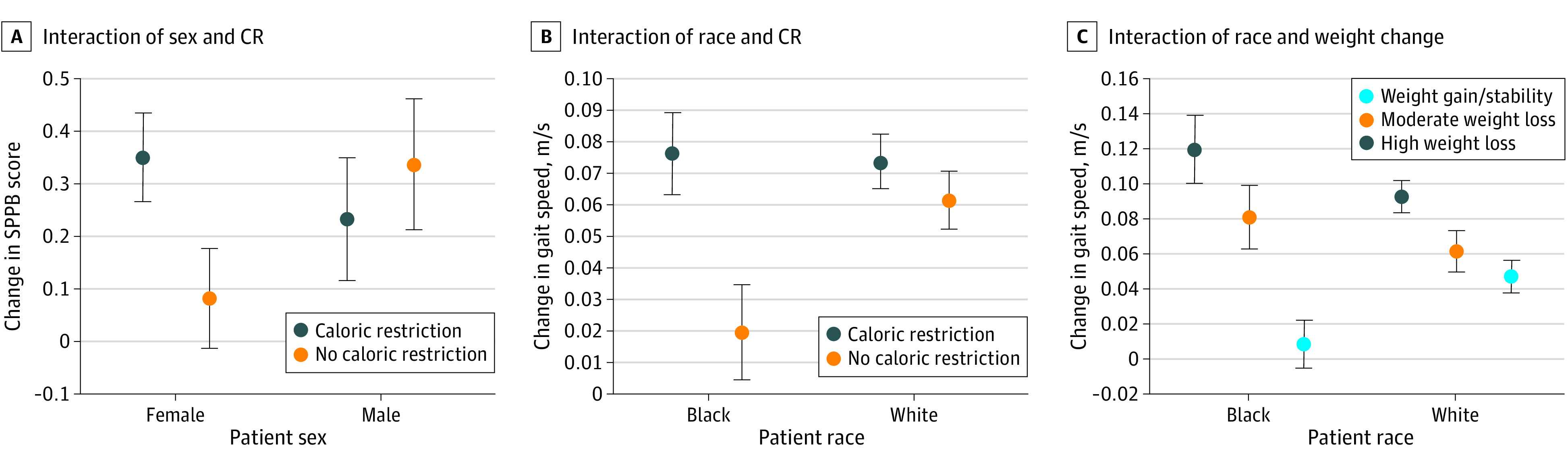
Sex- and Race-Stratified Associations Between Caloric Restriction (CR) or Weight Change Categories and Physical Function Data are shown when the interaction term was *P* < .05. Model statements are adjusted for age, sex, or race, as appropriate, and study, educational level, body mass index, comorbid status, and baseline value of the outcome. Error bars indicate 95% CI.

A sex by CR category interaction was observed for SPPB (*P* = .03), with women assigned to CR experiencing greater improvement in SPPB score (CR group, 0.35 [95% CI, 0.18-0.52]; non-CR group, 0.08 [95% CI, −0.11 to 0.27]) compared with men (CR group, 0.23 [95% CI, 0.00-0.46]; non-CR group, 0.34 [95% CI, 0.09-0.58]). A race by CR category interaction was observed for gait speed (*P* = .01), with Black participants assigned to CR experiencing greater improvement (CR group, 0.08 [95% CI, 0.05-0.10] m/s; non-CR group, 0.02 [95% CI, −0.01 to 0.05] m/s) compared with White participants (CR group, 0.07 [95% CI, 0.06-0.09] m/s; non-CR group, 0.06 [95% CI, 0.04-0.08] m/s). A race by weight change category interaction was also observed for gait speed (*P* = .006), with greater weight loss associated with greater improvement in White participants (high weight loss, 0.09 [95% CI, 0.07-0.11] m/s compared with weight gain/stability, 0.05 [95% CI, 0.03-0.07] m/s), although gains were most apparent in Black participants experiencing high weight loss (0.12 [95% CI, 0.08-0.16] m/s) compared with those experiencing weight gain/stability (0.01 [95% CI, −0.02 to 0.04] m/s). Sensitivity analysis revealed no significant 3-way interactions among treatment arm or weight change category, sex or race, and exercise assignment; likewise, further adjustment for exercise assignment did not significantly alter results (see eTable 3 in the Supplement).

Because body composition changes may underlie observed associations, exploratory models were fit with change in fat mass and lean mass as the outcome among the subset of individual with complete baseline and follow-up DXA data (n = 360). Across all CR groups, fat mass loss was similar. Lean mass loss was similar in White and Black participants; however, women lost more lean mass compared with men (−2.7 [95% CI, −2.2 to −3.2] vs −1.2 [95% CI, −0.3 to −2.0] kg).

## Discussion

Results from this analysis suggest that the functional benefit of weight loss in older adults may differ by sex and race. Specifically, weight loss–associated improvement in SPPB score was greater in women than men, and the beneficial effect of weight loss on gait speed was greater in Black than White participants and augmented with greater weight loss. Importantly, the degree of difference we observed between subgroups aligns with clinically meaningful thresholds (ie, 0.30-point change in SPPB^33^ and a 0.05-m/s change in gait speed^34^). This not only confers domain-specific pragmatic information to the geriatrician recommending weight loss to patients but also underscores the need to consider relevant biological variables—such as sex and race—in clinical research design.

A logical ensuing question from our results is, what is driving the differential treatment effects? Although inferential capability of our data set is limited, we draw on the larger literature to aid in interpretation. Observational data show women have greater susceptibility to obesity-associated disability than men^20^; therefore, greater weight loss–associated improvements in SPPB score in women could be expected, despite greater lean mass losses noted in our exploratory DXA analysis. Indeed, trial data in older adults demonstrate that fat mass loss is a more significant covariate associated with change in physical function than lean mass loss.^35^ In contrast, all men in the pooled analysis—regardless of weight loss assignment—experienced modest improvement in SPPB score, which suggests a different mechanism of action. Social engagement is an important determinant of functional status in older adults,^36^ with men typically reporting less social connectedness than women.^37^ Because included trials were behavioral based, opportunity for social engagement would have increased for all participants and could underlie the universal improvement in SPPB score we observed in men. Sex differences are also noted in the health benefits derived from exericse,^38^ with data pointing to greater muscle strength and quality gains experienced by men compared with women.^39^ Although we adjusted for exercise assignment, doing so affects the relative, not absolute, treatment responses. Thus, the residual effect of exercise may explain the observed SPPB improvement in men across CR categories.

Black participants are more likely to have^18^ or develop^19^ physical impairment than White participants for the same BMI; thus, as with women, greater weight loss–associated gait speed improvement in Black participants could be expected. Limited trial data examining the effects of weight loss on physical function in Black and White participants suggest similar functional improvement with similar weight loss^40^; however, the weight loss threshold beyond which functional benefit is conferred may differ. In the present analysis, Black individuals experienced less weight loss, yet greater improvement in gait speed compared with White individuals. Similarly, a systematic review of National Institutes of Health–funded, multicenter, behavioral lifestyle interventions also suggests the health effects of weight reduction are more profound for Black than White individuals, with Blacks experiencing greater improvement in cardiometabolic risk factors per unit of weight lost.^13^ Exploratory body composition results from the present analysis reveal similar absolute lean mass loss for both racial groups, despite larger absolute weight loss achieved in White vs Black participants, which may contribute to differential gait speed response. That said, improvement in gait speed was noted in White individuals regardless of CR assignment category. As with men and SPPB response, we speculate that this difference could be owing to social facilitation and/or exercise, because these variables were present in all categories, and we implore the larger research community to help explain the phenomenology presented in this report.

### Strengths and Limitations

Strengths of this study include the unique ability to generate a large sample by pooling individual-level data from randomized clinical trials with similar major design elements. In addition, standardized protocols were used to collect all physical function data (including training/certification of functional assessors and use of standardized script language), with gait speed and SPPB well represented across studies. Although some heterogeneity among the trials can be acknowledged as a limitation, it also broadens the generalizability of our findings and protects against overinterpretation of idiosyncratic results from any single study. Although weight loss is the central process measure of interest, we are unable to adjust for other measures of compliance, such as intervention attendance, which may also be important drivers of variability in treatment response. Similarly, we did not fully explore the effect of exercise on change in absolute physical function, although sensitivity analyses adjusting for exercise did not materially affect study findings. Finally, while our findings are provocative, they are certainly not definitive, and replication is warranted.

## Conclusions

This secondary analysis of 8 randomized clinical trials found that women and Black participants were more likely to experience functional benefit from a weight loss intervention than men or White participants. These findings affirm the need to consider relevant biological variables in clinical research—with the important caveat that this burden should not fall solely on individual investigators. Fundamentally, the problem is one of sample size and speaks to the need to have data sharing mechanisms in place to pool studies of similar interventions, as well as a repository of stratified results. Future work seeking to clarify the extent and correlates of interindividual variability to treatment response in additional scientific domains has major implications for patients, clinicians, and the larger scientific community.

## Notes

Supplement. **eTable 1.** Baseline Demographic Characteristics by Sex **eTable 2.** Baseline Demographic Characteristics by Race **eTable 3.** Type III Tests of 3-Way Interactions for Sensitivity Analyses Involving Exercise

## References

[1] Lauer M Consideration of relevant biological variables in NIH grant applications. NIH Office of Extramural Research. Posted January 29, 2016 Accessed December 12, 2019. https://nexus.od.nih.gov/all/2016/01/29/consideration-of-relevant-biological-variables-in-nih-grant-applications/

[2] Geller SE, Adams MG, Carnes M Adherence to federal guidelines for reporting of sex and race/ethnicity in clinical trials. J Womens Health (Larchmt). 2006;15(10):1123-1131. doi:10.1089/jwh.2006.15.1123 17199453

[3] Clayton JA Studying both sexes: a guiding principle for biomedicine. FASEB J. 2016;30(2):519-524. doi:10.1096/fj.15-279554 26514164 PMC4714546

[4] Burchard EG, Ziv E, Coyle N, The importance of race and ethnic background in biomedical research and clinical practice. N Engl J Med. 2003;348(12):1170-1175. doi:10.1056/NEJMsb025007 12646676

[5] National Institute on Aging. Interventions Testing Program (ITP). Cited January 21, 2020. Accessed January 21, 2020. https://www.nia.nih.gov/research/dab/interventions-testing-program-itp

[6] Clayton JA, Arnegard ME Taking cardiology clinical trials to the next level: a call to action. Clin Cardiol. 2018;41(2):179-184. doi:10.1002/clc.22907 29480590 PMC6489876

[7] Taylor AL, Wright JT Jr Should ethnicity serve as the basis for clinical trial design? importance of race/ethnicity in clinical trials: lessons from the African-American Heart Failure Trial (A-HeFT), the African-American Study of Kidney Disease and Hypertension (AASK), and the Antihypertensive and Lipid-Lowering Treatment to Prevent Heart Attack Trial (ALLHAT). Circulation. 2005;112(23):3654-3660. doi:10.1161/CIRCULATIONAHA.105.540443 16330707

[8] Woitowich NC, Woodruff TK Implementation of the NIH sex-inclusion policy: attitudes and opinions of study section members. J Womens Health. 2019;28(1):9-16. doi:10.1089/jwh.2018.7396 30539677

[9] Geller SE, Koch AR, Roesch P, Filut A, Hallgren E, Carnes M The more things change, the more they stay the same: a study to evaluate compliance with inclusion and assessment of women and minorities in randomized controlled trials. Acad Med. 2018;93(4):630-635. doi:10.1097/ACM.0000000000002027 29053489 PMC5908758

[10] Hales CM, Carroll MD, Fryar CD, Ogden CL Prevalence of obesity among adults and youth: United States, 2015-2016. NCHS Data Brief. 2017;(288):1-8. 29155689

[11] Pagoto SL, Schneider KL, Oleski JL, Luciani JM, Bodenlos JS, Whited MC Male inclusion in randomized controlled trials of lifestyle weight loss interventions. Obesity (Silver Spring). 2012;20(6):1234-1239. doi:10.1038/oby.2011.140 21633403

[12] Haughton CF, Silfee VJ, Wang ML, Racial/ethnic representation in lifestyle weight loss intervention studies in the United States: a systematic review. Prev Med Rep. 2018;9:131-137. doi:10.1016/j.pmedr.2018.01.012 29616185 PMC5880332

[13] Wingo BC, Carson TL, Ard J Differences in weight loss and health outcomes among African Americans and Whites in multicentre trials. Obes Rev. 2014;15(suppl 4):46-61. doi:10.1111/obr.12212 25196406

[14] Rejeski WJ, Marsh AP, Chmelo E, Rejeski JJ Obesity, intentional weight loss and physical disability in older adults. Obes Rev. 2010;11(9):671-685. doi:10.1111/j.1467-789X.2009.00679.x 19922431 PMC2888667

[15] Courtney-Long EA, Carroll DD, Zhang QC, Prevalence of disability and disability type among adults—United States, 2013. MMWR Morb Mortal Wkly Rep. 2015;64(29):777-783. doi:10.15585/mmwr.MM6429a2 26225475 PMC4584831

[16] Fried LP, Guralnik JM Disability in older adults: evidence regarding significance, etiology, and risk. J Am Geriatr Soc. 1997;45(1):92-100. doi:10.1111/j.1532-5415.1997.tb00986.x 8994496

[17] Houston DK, Nicklas BJ, Zizza CA Weighty concerns: the growing prevalence of obesity among older adults. J Am Diet Assoc. 2009;109(11):1886-1895. doi:10.1016/j.jada.2009.08.014 19857630

[18] Kirkness CS, Ren J Race differences: use of walking speed to identify community-dwelling women at risk for poor health outcomes—Osteoarthritis Initiative Study. Phys Ther. 2015;95(7):955-965. doi:10.2522/ptj.20140028 25655879 PMC4498144

[19] Wei L, Wu B Racial and ethnic differences in obesity and overweight as predictors of the onset of functional impairment. J Am Geriatr Soc. 2014;62(1):61-70. doi:10.1111/jgs.12605 24384026 PMC4296972

[20] Visser M, Langlois J, Guralnik JM, High body fatness, but not low fat-free mass, predicts disability in older men and women: the Cardiovascular Health Study. Am J Clin Nutr. 1998;68(3):584-590. doi:10.1093/ajcn/68.3.584 9734734

[21] Botoseneanu A, Allore HG, Mendes de Leon CF, Gahbauer EA, Gill TM Sex differences in concomitant trajectories of self-reported disability and measured physical capacity in older adults. J Gerontol A Biol Sci Med Sci. 2016;71(8):1056-1062. doi:10.1093/gerona/glw038 27071781 PMC4945890

[22] Messier SP, Loeser RF, Miller GD, Exercise and dietary weight loss in overweight and obese older adults with knee osteoarthritis: the Arthritis, Diet, and Activity Promotion Trial. Arthritis Rheum. 2004;50(5):1501-1510. doi:10.1002/art.20256 15146420

[23] Rejeski WJ, Brubaker PH, Goff DC Jr, Translating weight loss and physical activity programs into the community to preserve mobility in older, obese adults in poor cardiovascular health. Arch Intern Med. 2011;171(10):880-886. doi:10.1001/archinternmed.2010.522 21263080 PMC4425192

[24] Messier SP, Mihalko SL, Legault C, Effects of intensive diet and exercise on knee joint loads, inflammation, and clinical outcomes among overweight and obese adults with knee osteoarthritis: the IDEA randomized clinical trial. JAMA. 2013;310(12):1263-1273. doi:10.1001/jama.2013.277669 24065013 PMC4450354

[25] Nicklas BJ, Chmelo E, Delbono O, Carr JJ, Lyles MF, Marsh AP Effects of resistance training with and without caloric restriction on physical function and mobility in overweight and obese older adults: a randomized controlled trial. Am J Clin Nutr. 2015;101(5):991-999. doi:10.3945/ajcn.114.105270 25762810 PMC4409692

[26] Nicklas BJ, Brinkley TE, Houston DK, Effects of caloric restriction on cardiorespiratory fitness, fatigue, and disability responses to aerobic exercise in older adults with obesity: a randomized controlled trial. J Gerontol A Biol Sci Med Sci. 2019;74(7):1084-1090. doi:10.1093/gerona/gly159 29982294 PMC6580693

[27] Kitzman DW, Brubaker P, Morgan T, Effect of caloric restriction or aerobic exercise training on peak oxygen consumption and quality of life in obese older patients with heart failure with preserved ejection fraction: a randomized clinical trial. JAMA. 2016;315(1):36-46. doi:10.1001/jama.2015.17346 26746456 PMC4787295

[28] Normandin E, Yow D, Crotts C, Kiel J, Beavers KM, Nicklas BJ Feasibility of weighted vest use during a dietary weight loss intervention and effects on body composition and physical function in older adults. J Frailty Aging. 2018;7(3):198-203. doi:10.14283/jfa.2018.17 30095153 PMC6489119

[29] Beavers KM, Nesbit BA, Kiel JR, Effect of an energy-restricted, nutritionally complete, higher protein meal plan on body composition and mobility in older adults with obesity: a randomized controlled trial. J Gerontol A Biol Sci Med Sci. 2019;74(6):929-935. doi:10.1093/gerona/gly146 30629126 PMC6521917

[30] ATS Committee on Proficiency Standards for Clinical Pulmonary Function Laboratories ATS statement: guidelines for the six-minute walk test. Am J Respir Crit Care Med. 2002;166(1):111-117. doi:10.1164/ajrccm.166.1.at1102 12091180

[31] Simonsick EM, Montgomery PS, Newman AB, Bauer DC, Harris T Measuring fitness in healthy older adults: the Health ABC Long Distance Corridor Walk. J Am Geriatr Soc. 2001;49(11):1544-1548. doi:10.1046/j.1532-5415.2001.4911247.x 11890597

[32] Guralnik JM, Simonsick EM, Ferrucci L, A short physical performance battery assessing lower extremity function: association with self-reported disability and prediction of mortality and nursing home admission. J Gerontol. 1994;49(2):M85-M94. doi:10.1093/geronj/49.2.M85 8126356

[33] Kwon S, Perera S, Pahor M, What is a meaningful change in physical performance? findings from a clinical trial in older adults (the LIFE-P study). J Nutr Health Aging. 2009;13(6):538-544. doi:10.1007/s12603-009-0104-z 19536422 PMC3100159

[34] Perera S, Mody SH, Woodman RC, Studenski SA Meaningful change and responsiveness in common physical performance measures in older adults. J Am Geriatr Soc. 2006;54(5):743-749. doi:10.1111/j.1532-5415.2006.00701.x 16696738

[35] Beavers KM, Miller ME, Rejeski WJ, Nicklas BJ, Kritchevsky SB Fat mass loss predicts gain in physical function with intentional weight loss in older adults. J Gerontol A Biol Sci Med Sci. 2013;68(1):80-86. doi:10.1093/gerona/gls092 22503993 PMC3598365

[36] Dombrowsky TA Relationship between engagement and level of functional status in older adults. SAGE Open Med. 2017;5:2050312117727998. doi:10.1177/2050312117727998 28904793 PMC5588797

[37] McKenzie SK, Collings S, Jenkin G, River J Masculinity, social connectedness, and mental health: men’s diverse patterns of practice. Am J Mens Health. 2018;12(5):1247-1261. doi:10.1177/1557988318772732 29708008 PMC6142169

[38] Hands B, Parker H, Larkin D, Cantell M, Rose E Male and female differences in health benefits derived from physical activity: implications for exercise prescription. J Womens Health Issues Care. 2016;2016. doi:10.4172/2325-9795.1000238

[39] Da Boit M, Sibson R, Meakin JR, Sex differences in the response to resistance exercise training in older people. Physiol Rep. 2016;4(12):e12834. doi:10.14814/phy2.12834 27354538 PMC4923234

[40] Anton SD, Manini TM, Milsom VA, Effects of a weight loss plus exercise program on physical function in overweight, older women: a randomized controlled trial. Clin Interv Aging. 2011;6:141-149. doi:10.2147/CIA.S17001 21753869 PMC3131984

