# Error in Description of Race/Ethnicity

**DOI:** 10.1001/jamanetworkopen.2020.23164

**Published:** 2020-09-21

In the Original Investigation titled “Association of Sex or Race With the Effect of Weight Loss on Physical Function: A Secondary Analysis of 8 Randomized Clinical Trials,”^1^ published online August 21, 2020, the term “biological” was incorrectly used to describe differences in participant race/ethnicity in the Key Points, Abstract, Discussion, and Conclusion. This article has been corrected.
