# Supplementary material for: Association of Sex or Race With the Effect of Weight Loss on Physical Function: A Secondary Analysis of 8 Randomized Clinical Trials
Source: JAMA Netw Open. 2020 Aug 21;3(8):e2014631. doi: 10.1001/jamanetworkopen.2020.14631 (PMC7442923; doi:10.1001/jamanetworkopen.2020.14631)
Supplement: Supplement. — eTable 1. Baseline Demographic Characteristics by Sex eTable 2. Baseline Demographic Characteristics by Race eTable 3. Type III Tests of 3-Way Interactions for Sensitivity Analyses Involving Exercise [file jamanetwopen-3-e2014631-s001.pdf]

Supplementary Online Content

Beavers KM, Neiberg RH, Kritchevsky SB, et al. Association of sex or race with the effect of weight loss on physical function: a secondary analysis of 8 randomized clinical trials. *JAMA Netw Open*. 2020;3(8): e2014631. doi:10.1001/jamanetworkopen.2020.14631

eTable 1. Baseline Demographic Characteristics by Sex

eTable 2. Baseline Demographic Characteristics by Race

eTable 3. Type III Tests of 3-Way Interactions for Sensitivity Analyses Involving Exercise

This supplementary material has been provided by the authors to give readers additional information about their work.

©2020 Beavers KM et al. *JAMA Network Open*.

eTable 1. Baseline Demographic Characteristics by Sex

**Variable**	**Overall (N=1317)**	**Male (N=397)**	**Female (N=920)**	***p*-value**
Age (years)	67.7 ± 5.4	68.0 ± 5.0	67.5 ± 5.5	0.10
Caloric Restriction Assignment, n (%)	734 (55.7)	213 (53.7)	521 (56.6)	0.32
Race, n (%)				<0.001
White	1042 (79.1)	360 (90.7)	682 (74.1)	
Black	275 (20.9)	37 (9.3)	238 (25.9)	
Education, n (%)				<0.001
Primary/Secondary Only	255 (19.4)	68 (17.1)	187 (20.3)	
College Graduate	780 (59.2)	213 (53.7)	567 (61.6)	
Post College Graduate	282 (21.4)	116 (29.2)	166 (18.0)	
BMI (kg/m²)	33.9 ± 4.4	33.5 ± 3.7	34.1 ± 4.7	0.02
Comorbidities, n (%)				
Diabetes	188 (14.3)	55 (13.9)	133 (14.5)	0.77
Hypertension	768 (58.3)	236 (59.4)	532 (57.8)	0.58
CVD History	448 (34.0)	151 (38.0)	297 (32.3)	0.04
Physical Function Assessments				
Fast Gait Speed (m/s)	1.2 ± 0.2	1.3 ± 0.2	1.2 ± 0.2	<0.001
SPPB (0-12 score)	10.4 ± 1.5	10.7 ± 1.3	10.3 ± 1.5	<0.001
DXA Body Composition Measures				
Total Fat Mass (kg)	38.0 ± 8.7	33.8 ± 7.9	39.8 ± 8.3	<0.001
Total Lean Mass (kg)	53.2 ± 11.1	67.0 ± 7.3	47.2 ± 6.0	<0.001
Percentage Body Fat (%)	40.7 ± 7.1	32.4 ± 4.8	44.4 ± 4.2	<0.001

Data presented as mean ± SD or n (%). Abbreviations: BMI = body mass index; CVD = cardiovascular disease; DXA = dual energy x-ray absorptiometry; SPPB = short physical performance battery. Baseline samples sizes associated with individual outcome measures are as follows: Fast gait speed n=1245, SPPB n=853, and DXA-acquired body composition n=360.

©2020 Beavers KM et al. *JAMA Network Open*.

eTable 2. Baseline Demographic Characteristics by Race

Variable	Overall (N=1317)	White (N=1042)	Black (N=275)	*p*-value
Age (years)	67.7 ± 5.4	67.7 ± 5.3	67.6 ± 5.5	0.78
Caloric Restriction Assignment, n (%)	734 (55.7)	574 (55.1)	160 (58.2)	0.36
Sex, n (%)				<0.001
Female	920 (69.9)	682 (65.5)	238 (86.5)	
Male	397 (30.1)	360 (34.5)	37 (13.5)	
Education, n (%)				0.80
Primary/Secondary Only	255 (19.4)	198 (19.0)	57 (20.7)	
College Graduate	780 (59.2)	619 (59.4)	161 (58.5)	
Post College Graduate	282 (21.4)	225 (21.6)	57 (20.7)	
BMI (kg/m^2)	33.9 ± 4.4	33.4 ± 4.0	36.0 ± 5.1	<0.001
Comorbidities				
Diabetes	188 (14.3)	122 (11.7)	66 (24.0)	<0.001
Hypertension	768 (58.3)	565 (54.2)	203 (73.8)	<0.001
CVD History	448 (34.0)	350 (33.6)	98 (35.6)	0.52
Physical Function Assessments				
Fast Gait Speed	1.2 ± 0.2	1.3 ± 0.2	1.1 ± 0.2	<0.001
SPPB (0-12 score)	10.4 ± 1.5	10.5 ± 1.5	10.1 ± 1.5	0.001
DXA Body Composition Measures				
Total Fat Mass (kg)	38.0 ± 8.7	37.2 ± 8.1	41.1 ± 10.1	<0.001
Total Lean Mass (kg)	53.2 ± 11.1	53.5 ± 11.6	52.0 ± 8.9	0.05
Percentage Body Fat (%)	40.7 ± 7.1	40.2 ± 7.0	42.8 ± 7.0	<0.001

Data presented as mean ± SD or n (%). Weight change categories are defined as: Weight Gain/Stability: <3% loss; Moderate Weight Loss: 3% to 7%; High Weight Loss: ≥7%. Abbreviations: BMI = body mass index; CVD = cardiovascular disease; DXA = dual energy x-ray absorptiometry; SPPB = short physical performance battery. Baseline samples sizes associated with individual outcome measures are as follows: Fast gait speed n=1245, SPPB n=853, and DXA-acquired body composition n=360.

©2020 Beavers KM et al. *JAMA Network Open*.

eTable 3. Type III Tests of 3-Way Interactions for Sensitivity Analyses Involving Exercise

**Six-Month Outcome**	**Interaction Variables**	**Three-way Interaction *p*-value**
SPPB Change	CR, Exercise, Sex	0.20
	CR, Exercise, Race	0.55
	% Weight Change, Exercise, Sex	0.67
	% Weight Change, Exercise, Race	0.72
Gait Speed Change	CR, Exercise, Sex	0.96
	CR, Exercise, Race	0.52
	% Weight Change, Exercise, Sex	0.77
	% Weight Change, Exercise, Race	0.99

Abbreviations: SPPB – Short Physical Performance Battery; CR – Assignment to Caloric Restriction. All models contain main effects of the stated interaction variables (3 terms), 2-way interactions (3 terms), and further adjustments for sex, age, race, study, education, BMI at baseline, diabetes history, hypertension history, cardiovascular disease history, and baseline values of the respective outcome. % Weight Change is modeled as a categorical variable, with weight gain/stability, moderate weight loss, and high weight loss categories represented.

©2020 Beavers KM et al. *JAMA Network Open*.
